# Who Accepts and Who Uses Community-Based Secondary Distribution HIV Self-Testing (HIVST) Kits? Findings From the Intervention Arm of a Cluster-Randomized Trial of HIVST Distribution Nested in Four HPTN 071 (PopART) Communities in Zambia

**DOI:** 10.1097/QAI.0000000000002344

**Published:** 2020-03-13

**Authors:** Bernadette Hensen, Albertus J. Schaap, Chama Mulubwa, Sian Floyd, Kwame Shanaube, Mwelwa M. Phiri, Virginia Bond, Chiti Bwalya, Musonda Simwinga, Sarah Fidler, Richard Hayes, Alwyn Mwinga, Helen Ayles

**Affiliations:** a Clinical Research Department, London School of Hygiene and Tropical Medicine, London, United Kingdom;; b Department of Infectious Disease Epidemiology, London School of Hygiene and Tropical Medicine, London, United Kingdom;; c Zambart, Lusaka, Zambia;; d Department of Global Health and Development, London School of Hygiene and Tropical Medicine, London, United Kingdom; and; e Imperial College, London, United Kingdom and Imperial College NIHR BRC.

**Keywords:** HIV self-testing, men, HIV testing, Zambia, sub-Saharan Africa

## Abstract

HPTN 071 (PopART) was a community-randomized trial of a universal testing-and-treatment intervention on HIV incidence at population level in Zambia and South Africa. In Zambia, a trial of community-based distribution of HIV self-testing (HIVST) kits, including secondary distribution, as an option for HIV-testing was nested within 4 PopART intervention communities. We used data from the intervention arm of the nested trial to measure levels of and factors associated with acceptance and use of secondary distribution HIVST kits.

**Methods:**

Community HIV care providers offered the PopART combination HIV-prevention intervention door-to-door, systematically visiting all households and enumerating all household members. From 1 February to 30 April 2017, individuals aged 16 years and older consenting to PopART were offered the option to HIV self-test, if eligible for HIV-testing services. Individuals aged 18 years and older who reported a partner absent during household visits were offered an HIVST kit for secondary distribution to this partner. We used two data sources to measure acceptance and use of secondary distribution HIVST kits.

**Results:**

Among 9105 individuals aged 18 years and older consenting to PopART, 9.1% (n = 825) accepted an HIVST kit for secondary distribution. Approximately 55.8% reported that the kit had been used. Women were more likely to accept, and men more likely to use, secondary distribution HIVST kits. Kits were more likely to be used by individuals aged 30+ and who had not participated in a previous round of PopART. Approximately 6.8% had a reactive result.

**Conclusions:**

Community-based secondary distribution of HIVST kits reached men absent during community HIV care provider household visits and is a complement to facility- and community-based HIV-testing services, which often miss men.

## INTRODUCTION

Testing for HIV facilitates entry into the treatment continuum and access to prevention services. Despite its centrality to service access, in 2017, one-quarter of individuals remained unaware of their HIV-positive status globally.^1^ In sub-Saharan Africa, men are among those not being reached by HIV services. Of individuals living with HIV, ∼58% of boys/men know their HIV-positive status compared with 78% of girls/women.^2^ Drivers of men's low uptake of HIV-testing services are multifaceted, including health system and economic factors, and masculinity norms.^3–5^ As a consequence, HIV-positive men experience higher morbidity and mortality than HIV-positive women,^2^ and risk onward transmission to sexual partners.^6–8^ In Zambia in 2015/16, viral load suppression among individuals aged 25–34 was >50% among women and ∼35% among men.^2,9^ Reducing morality and reaching the UNAIDS 90-90-90 targets of zero HIV transmission requires a concerted effort to reach everyone with HTS, including men.

Since 2016, WHO has supported the implementation of evidence-based strategies to deliver HIV self-testing (HIVST).^10–13^ Through increased autonomy about when and where to test, HIVST has the potential to increase HIV-testing coverage among men and other groups not reached by available HTS. Secondary distribution of HIVST, where an HIVST kit is given to an individual for distribution to a third party, has been shown to reach male partners of women attending health facilities and can facilitate couples-testing.^14,15^ To date, there is little evidence on community-based secondary distribution of HIVST.

HPTN 071 (PopART) was a community-randomized trial of a universal testing-and-treatment (UTT) intervention on HIV incidence at population level.^16^ After 2 years of service delivery in Zambia, PopART reached the first UNAIDS 90% target among HIV-positive women, approaching it among men.^17^ A challenge was reaching men aged 25–54 years, who were often absent during home-based service delivery.^17,18^ To address this gap, a cluster-randomized trial (CRT) of HIVST as an alternative choice for how to test for HIV was nested in 4 Zambian PopART communities.^19^ Overall, the HIVST intervention showed a small but significant increase in knowledge of HIV status among men.^20^ As part of the HIVST intervention, individuals whose partner (a household member who is a spouse/cohabiting partner) was absent during lay counselor's household visits were offered an HIVST kit for distribution to this partner. This strategy primarily aimed to reach men^18^ and support couples-testing. Analysis of the primary outcome found that 14% of men who self-tested did so using a secondary distribution HIVST kit.^20^

Using data from the nested HIVST trial, we describe characteristics of individuals who accepted an HIVST kit for secondary distribution and, among these individuals, describe characteristics associated with reporting use of the kit. We describe return of secondary distribution HIVST kits and characteristics of individuals who used these kits. We use findings to provide recommendations for implementation of secondary distribution outside of health facilities as part of a UTT strategy.

## METHODS

### Study Location and Population

HPTN 071 (PopART) was conducted from 2014 to 2017 in South Africa and Zambia. In Zambia, HPTN 071 (PopART) was implemented in 12 communities, defined as the catchment population of a government primary health care facility.^16^ The HIVST CRT was nested in 4 communities that were randomized to receive the PopART intervention. Communities randomized to receive the PopART intervention were divided into geographical “zones” of ∼450–500 households. Within each zone, a pair of trained lay counselors, called community HIV care providers (CHiPs), delivered PopART systematically to each household within their zone.^16^ In the 4 PopART communities included in the HIVST nested trial, restricted randomization was used to allocate 66 zones to either rapid HIV-testing on a finger-prick blood sample performed by a CHiP (PopART standard of care) or the PopART intervention plus the offer of HIVST.^20^

Details of the nested CRT are published elsewhere.^20^ Briefly, all individuals aged 16 years and older, resident in a household in the 4 communities and enumerated during the HIVST intervention (February 1-April 30, 2017, during a third round of PopART) were eligible to participate in the HIVST trial.^16,20^ For this analysis, we used data only from 33 zones allocated to the PopART plus HIVST intervention.

### The PopART Plus HIVST Intervention

The PopART intervention included door-to-door delivery of HTS with referral to care and immediate treatment for individuals testing HIV-positive or to prevention services for individuals testing HIV-negative.^16^ During household visits, CHiPs used electronic data capture (EDC) devices to systematically enumerate all households within their zones and collect data on services offered to and used by household members consenting to PopART.^18,21^ CHiPs offered HTS to individuals who reported that their last HIV test was negative or that they had never HIV tested. During the HIVST intervention period, when offering HTS to eligible individuals, CHiPs offered the option of either finger-prick HIV testing or oral HIVST.^20^ Individuals choosing HIVST could choose supervised HIVST in the presence of the CHiP or unsupervised HIVST.^20^

**FIGURE 1.**
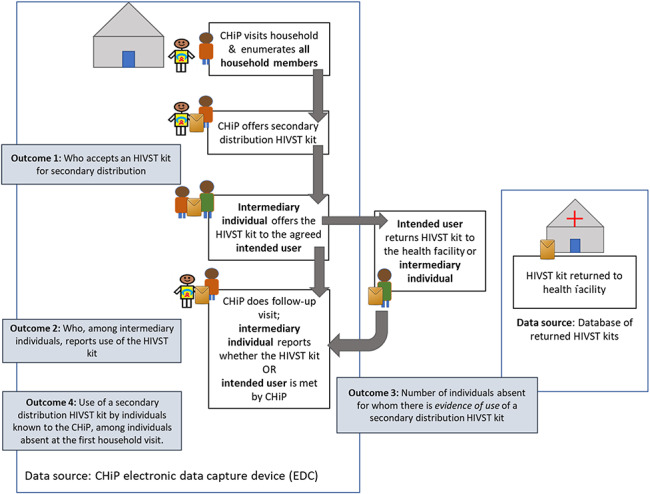
Schematic illustrating the distribution and return of secondary distribution HIVST kits.

For individuals aged 18 years and older who consented and whose spouse/partner was absent at the time of the household visit, CHiPs offered to leave HIVST kit(s) for secondary distribution to this partner (the intended user) or for couples-testing with this partner (Fig. 1). CHiPs informed intermediary individuals, defined as individuals accepting a secondary distribution HIVST kit, that they should provide the intended user with information on how to perform and read the self-test and not force them to use the self-test.^24^ CHiPs also informed intermediary individuals that used (or unused) kits could be returned either to the CHiP or dropped off confidentially at the clinic. CHiPs left a card with contact details for additional support if needed. Within 7 days of leaving an HIVST kit, CHiPs conducted a follow-up visit. If the intended user was absent during follow-up, the intermediary individual was asked about use and results of the self-test. Data from follow-up visits were collected until 30 June 2017.

The HIVST package included an OraQuick kit, sealable envelope, and self-completed results (SCR) form, which asked age, sex, the self-test result, and questions on ease of kit use. The envelope intended to facilitate confidential return of HIVST kits and SCR forms. At the health facility, the study team installed a box where the envelope could be dropped off. All 3 items were labeled with the same unique barcode to allow the SCR form, the HIVST kit, and data on the intermediary individual who collected the secondary distribution HIVST kit to be linked.

A database of returned HIVST kits was developed to monitor kit return. This database included data on every HIVST kit returned to CHiPs/health facilities, including the HIVST barcode, whether the test had been used and the information available on the SCR form.

### Data Collection

The EDC used by CHiPs was modified to collect data for analysis of the primary outcome of the nested HIVST trial.^21^ Relevant to this analysis, modifications allowed CHiPs to record: whether an individual accepted an HIVST kit for secondary distribution, the unique barcode of the kit, and the intermediary individual's report of kit use. For each household member, data were available on whether the household member was absent at the time of a CHiP household visit and/or had used a secondary distribution HIVST kit. There were no data on the relationship between household members and so no data on the number of individuals consenting to PopART who had a partner absent during the CHiP household visit.

In their EDC, the CHiP was expected to record the result of the secondary distribution HIVST kit CHiP, if the intermediary individual reported that the kit had been used by an enumerated household member and they knew the result, to facilitate follow-up visits for HIV-related services, including confirmatory HTS.

### Study Outcomes

This analysis includes 4 outcomes. The first is the number and percentage of household members accepting an HIVST kit for secondary distribution (numerator) among individuals aged 18 years and older who consented to PopART (denominator). The second is the number and percentage of intermediary individuals who reported use of the HIVST kit, defined as reporting that the kit was used and/or reporting the self-test result to the CHiP (numerator) among the number of household members accepting an HIVST kit for secondary distribution (denominator).

The third outcome is the number and percentage of individuals for whom there is evidence that a secondary HIVST kit was used (numerator), among the denominator of all individuals absent at the first CHiP household visit (*evidence of use* outcome). This outcome used both data sources and was defined as either the HIVST kit returned used with a result entered on the SCR (recorded as negative, reactive, or indeterminate) or a secondary distribution HIVST kit result recorded by the CHiP (as reported by the intermediary individual or the intended user, if seen by the CHiP).

The fourth outcome is the number and percentage of individuals who used a secondary distribution HIVST kit and were enumerated by, and therefore known to, the CHiP (numerator), among individuals absent at the first CHiP household visit (denominator). In contrast to outcome 3, this outcome used EDC data only. The numerator included only individuals who had a secondary distribution HIVST kit result recorded by the CHiP.

Of note, the number of kits reported as used by the intermediary individual (outcome 2) is higher than the number of household members with a result reported to the CHiP (outcome 4). There are 3 possible reasons for this discrepancy: (1) the user of the kit was not a household member and not enumerated by the CHiP, (2) although the intermediary individual knew the kit had been used, they may not have known the result, or (3) the CHiP did not record the result in their EDC despite it being reported to them.

### Data Limitations

The EDC system had been used by CHiPs for 3 years. Changes to the system for this brief HIVST study were kept minimal. As such, and as PopART was a UTT strategy, household enumeration was not modified to record relationships between individuals within households. Data on the number of household members with a partner absent at the first CHiP household visit were not available.

Despite minimal changes, some CHiPs experienced challenges in data entry. Our analysis revealed that an intermediary individual might be recorded as accepting an HIVST kit for secondary distribution, yet there was no record of any absent household member; alternatively, a result for a secondary distribution HIVST kit was recorded for an individual but with no record of an intermediary individual in the household accepting a secondary distribution HIVST kit.

CHiPs recorded secondary distribution HIVST kit results for 323 household members.^20^ For 47 (14.6%; n = 47/323), there was no record of an intermediary individual who accepted a secondary distribution HIVST kit. Thirteen of these individuals had performed supervised HIVST with the CHiP and 2 self-reported their HIV-positive status. Adjusting for these individuals gave 276 individuals with a secondary distribution HIVST kit result, who were known to the CHiP and linked to an intermediary individual.

### Statistical Analysis

We describe and present in a flowchart the number of intermediary individuals and the number of individuals who report that the HIVST kit was used, not yet used (but the kit was expected to be used), or not used (and the kit was not expected to be used). We subsequently used the EDC and return of HIVST kit data to describe the number of secondary distribution HIVST kits that were returned and the number of kits for which there was evidence of use.

We next describe all outcomes and, for outcomes one and 2, explore the association between the intermediary individual's age, sex, previous residence in the CHiP zone, HIV status as recorded by the CHiP, previous participation in PopART and community of residence, and the outcomes. We also explored whether uptake of an offer of HTS and the type of HIV testing were associated with outcomes. For outcome 4, we explored the association between age, sex, previous residence in the CHiP zone, HIV status as recorded by the CHiP, previous participation in PopART, and community of residence of individuals absent at first household visit and the outcome. We used population-averaged models, allowing for clustering by zones and adjusting a priori for age group and sex, and for community to explain some of the between-zone variation, to investigate these associations.

## RESULTS

CHiPs recorded 834 household members as intermediary individuals. For 26 of these individuals, the kit was used by a household member present at the time of the CHiP's visit. These were not counted as secondary distribution in subsequent analysis. We also identified 47 individuals who had a result for a secondary distribution HIVST kit recorded, but no household member seen by the CHiP was listed as an intermediary individual. For 17, we were able to use household enumeration data to identify the intermediary individual likely to have accepted the secondary distribution HIVST kit.

**FIGURE 2.**
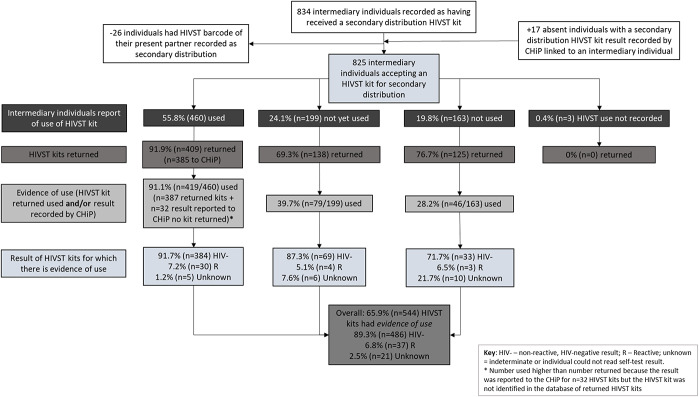
Flowchart describing the number and percent of intermediary individuals who reported use of secondary distribution HIVST kits, HIVST kits returned and used.

After these adjustments, 825 individuals were defined as intermediary individuals (Fig. 1) among whom 55.8% (n = 460/825) reported use and/or the result of the secondary distribution HIVST kit to the CHiP (Fig. 2). Twenty-eight-percent (n = 129/460; 28.0%) of these intermediary individuals were recorded as having received counseling as a couple or household.

One-quarter of intermediary individuals reported that the kit had not yet been used (n = 199/825; 24.1%), 19.8% (n = 163/825) that the kit would not be used, and for 0.4% (n = 3/825), data on use were missing. Of the 362 HIVST kits that intermediary individuals reported were not yet or would not be used, 34.5% (n = 125) were returned used (Fig. 2). For 102 (12.4%; n = 102/825) kits, there was no report that the kit had been used nor were the kits identified in the returned HIVST kit database.

Overall, there was evidence of use for 65.9% (n = 544) of the 825 secondary distribution HIVST kits (Fig. 2). The majority of kits were used by men (n = 448/541; 82.8%) and individuals aged 30 years and older (n = 368/534; 68.9%; Tables 3 and 4); overall, 6.8% (n = 37/544) of kits had a reactive result recorded by the CHiP or on the SCR form; of these kits, 6 (16.2%) intended users were subsequently seen by the CHiP.

### Outcomes 1 and 2: Acceptance and Reported Use of a Secondary Distribution HIVST Kit by Intermediary Individuals

Among 9105 individuals aged 18 years and older residing in zones randomized to the HIVST intervention and consenting to participate in PopART, 9.1% (n = 825) accepted an HIVST kit for secondary distribution. Women (n = 738/5428; 13.6%) were more likely than men (n = 87/3677; 2.4%) to be intermediary individuals [adjusted odds ratio (adjOR) = 7.02 95% confidence interval (CI): 5.07 to 9.72]. Acceptance of a secondary distribution HIVST kit was highest among household members aged 25–39. Two percent (n = 17/942; 1.8%) of individuals self-reporting their HIV-positive status were intermediary individuals compared to 9.9% (n = 808/8163) of individuals who reported never-testing or that their last HIV-test result was negative. Among individuals accepting an offer of HTS, individuals opting for unsupervised HIVST (n = 146/488; 29.9%) were more likely to be intermediary individuals relative to those choosing finger-prick HTS (n = 137/3154; 4.3%, adjOR = 10.56 95% CI: 7.30 to 15.27; Table 1).

**TABLE 1.**
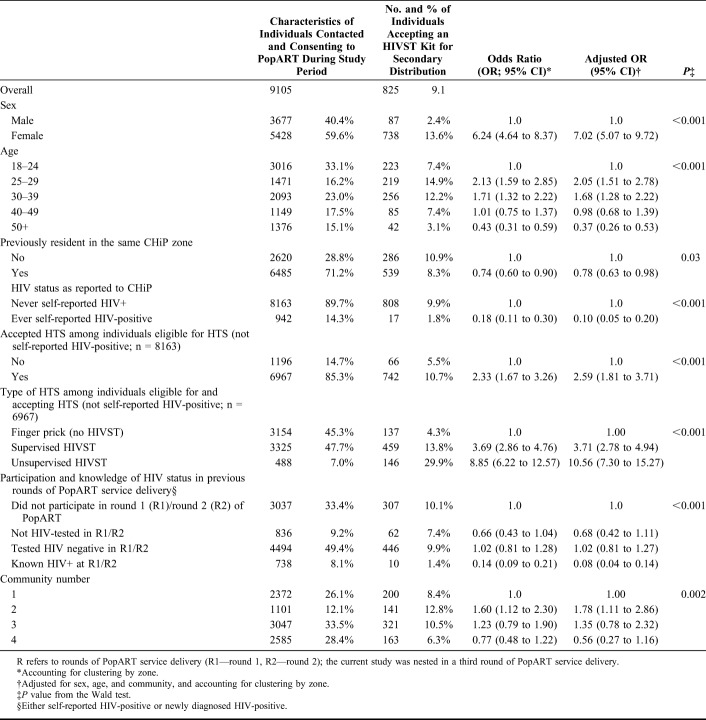
Characteristics of Individuals Aged 18 Years and Older Contacted and Consenting to Participate in PopART (N = 9105) and Factors Associated With Accepting an HIV Self-Testing Kit for Secondary Distribution (Outcome 1; n = 825)

Intermediary individuals younger than 40 years and who accepted an offer of HTS themselves were more likely to report that the secondary distribution HIVST kit had been used (Table 2). Among intermediary individuals who accepted an offer of HTS themselves, those choosing unsupervised HIVST were more likely to report use than individuals who chose finger-prick HTS for themselves.

**TABLE 2.**
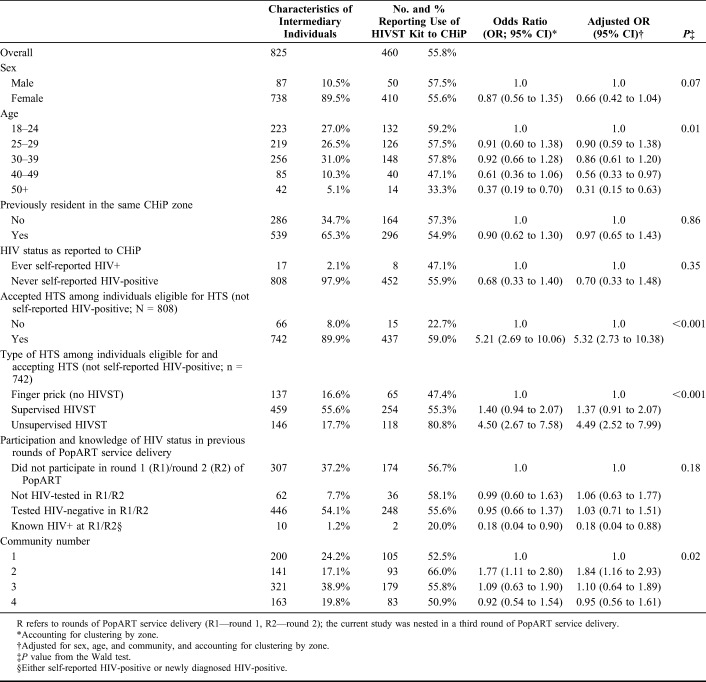
Characteristics of Intermediary Individuals (N = 825) Who Reported That the Secondary Distribution HIV Self-Test (HIVST) Kit had Been Used (Outcome 2; n = 460)

### Outcome 3: Evidence of Use of Secondary Distribution HIVST Kits

Forty-five percent of enumerated individuals (n = 5930/13,267; 44.7%) were absent at the first CHiP household visit. Using the definition of evidence of use, 9.2% (n = 544/5930) of individuals absent at the first household visit were estimated to have used a secondary distribution HIVST kit.

### Outcome 4: Use of Secondary Distribution HIVST Kits by Individuals Known to CHiPs

Among individuals enumerated by CHiPs but absent at the first household visit, men (0.9% among women vs 6.9% among men adjOR = 0.14, 95% CI: 0.09 to 0.21), individuals aged 30 years and older (adjOR = 2.00 95% CI: 1.38 to 2.90), previously not resident in the CHiP zone, and who did not participate in a second round of PopART were more likely to use secondary distribution HIVST kits (Tables 3 and 4).

**TABLE 3.**
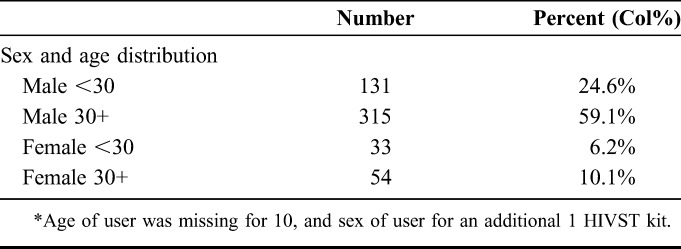
Sex and Age (N = 533) Distribution of Individuals Who Used a Secondary Distribution HIV Self-Testing Kit* (Outcome 4)

**TABLE 4.**
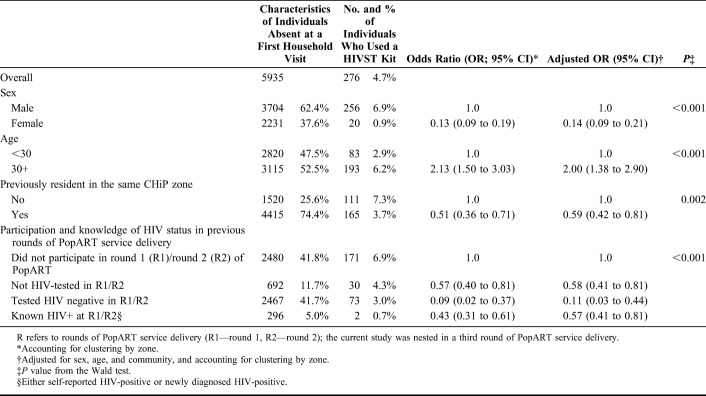
Use of a Secondary Distribution HIVST Kit by Individuals Known to CHiPs (n = 276) Among Individuals Absent at the First Household Visit (Outcome 4; N = 5935)

## DISCUSSION

Community-based secondary distribution HIVST kits were used by men and reached individuals who previously had not participated in PopART. Although many secondary distribution HIVST kits were returned, some were unused while, for others, use was unknown. Few individuals with a reactive self-test were subsequently seen by the CHiP during the study period. Secondary distribution strategies that are targeted at key demographic groups may minimize waste and increase reach and should be considered as part of a UTT strategy.

This is one of the first studies of secondary distribution outside of health facility settings and adds to the evidence base that the strategy is acceptable and can reach groups not reached through home-based HTS. In 2 studies in Kenya, health care workers offered female sex workers attending drop-in clinics^15^ and women attending antenatal or postpartum clinics an HIV self-test to distribute to sexual partners or other individuals.^14,15^ The trial reported a significant increase in partner-testing relative to invitations for clinic-based HTS.^14^ Men resident in zones included in this analysis reported that avoiding queues and “being seen” at the clinic were advantages of secondary distribution, in addition to allowing men to test at convenient times.^23^ Studies have shown that (non)-monetary costs associated with accessing facility-based HTS is particularly high for men.^5,24^ Community-based secondary distribution of HIVST overcame some of these barriers and is important in settings where men are under-reached.

Some secondary distribution HIVST kits were returned unused or their use was unknown. At present, HIVST kits are more expensive than rapid finger-prick HIV-tests. To reduce costs, targeted secondary distribution should be implemented.^25^ In antenatal care, this could be secondary distribution to women whose partner does not respond to invitations for HTS at health facilities. Outside of health facilities, this could be distribution to individuals accessing community-based HTS who report a partner whose HIV status they do not know or who last tested HIV-negative >12 months ago or through social networks. To encourage kit return and support linkage to services, nonfinancial incentives or nudges or use of SMS reminders could be explored. Fewer HIV-positive individuals had an absent household member, and few accepted a secondary distribution HIVST. With evidence that secondary distribution through index antiretroviral therapy patients increased partner-testing in Malawi,^26^ community-based secondary distribution strategies should offer more support for HIV-positive individuals to accept secondary distribution HIVST kits.

Measuring use of HIVST kits, which are intended for use in private, is challenging particularly with secondary distribution. In the 2 Kenyan studies, outcomes were reported by intermediary individuals.^14,15^ Our evidence of use measure combined individual report and data on HIVST kit return. Data from individuals only would have underestimated use. Underreporting of kit use by intermediary individuals may reflect timing of the CHiP visit, with the CHiP visiting the household before the test had been used, but also reflect the intended user's decision not to share information on use of the self-test kit. A longer reporting period might have seen the intermediary report be more reflective of returned test kits. Alternative methods for measuring use and facilitating HIVST kit return, particularly where HIVST is through secondary distribution, need to be explored if higher HTS uptake is to be translated into increased coverage of treatment and prevention services. Providing alternative ways for individuals to return kits and use of barcodes is recommended, as these allowed us to link kits to information on distribution and use as reported by intermediary individuals. Use of mobile phones to report HIVST kit use and provide information on prevention and care services should also be explored. A small number of individuals with a reactive result were seen by the CHiP during the study. A longer follow-up period for this analysis may have seen more individuals with a reactive HIVST followed-up by CHiPs, who were familiar with the communities and likely knew the intended users. Future research should collect data on who the intended user is to allow researchers to understand who is not being reached or not linked to care through secondary distribution.

At the time of implementation, HIVST was novel. The implementation period may have been too short to allow communities to adapt to HIVST.^20^ While CHiPs had been offering HTS for ∼3 years, HIVST was also new to CHiPs, with secondary distribution particularly novel. CHiPs may have felt that secondary distribution relinquished their role as lay counselors. The strategy proved labor intensive, requiring detailed explanations on how to explain HIVST to a third party. A longer implementation period may have seen more kits distributed for secondary use.

Our data were subject to limitations. As mentioned, we did not have data on the number of individuals consenting to PopART who, at the first CHiP household visit, had a partner that was absent. As such, our analysis underestimates acceptance of secondary distribution HIVST kits among individuals with an absent partner and likely underestimates reach among absent partners. Some CHiPs struggled with data entry for secondary distribution. We accounted for these in the analysis, yet some individuals with a secondary distribution HIVST kit result were not linked to an intermediary individual, particularly if they resided in large households, as more assumptions about relationships within households would have been required. Using data on returned kits, we were strict in our definition of evidence of use, excluding kits recorded as used but without an SCR form. In excluding these kits, our study may underestimate kits for which there is evidence of use. Data were primarily self-reported, and because of the nature of HIVST, we cannot be sure who used the kit. Rigorous measurement of use of HIVST kits remains a challenge.

The EDC system collected data on service delivery and uptake. Although these data presented some challenges for this analysis, data were collected systematically on all households and are a valuable resource to understand intervention coverage among a large population. The database on returned HIVST kits was not developed with research in mind, yet provided valuable information to complement self-report. Additional strengths are: we have been transparent about challenges and assumptions, highlight the importance of program data and opportunities for learning where such data are used strategically.^27^ Findings are generalizable to high prevalence HIV settings with regular exposure to HTS.

## CONCLUSIONS

Community-based secondary distribution HIVST kits were used by men and those older than 30 years, who are missed by current HTS.^2^ To increase reach and better understand service use, targeted secondary distribution strategies need to be developed and tested, including distribution through social networks, and innovative ways of encouraging test-kit return explored. To improve targeting, existing data on HTS uptake through different strategies need to be consolidated to understand key demographic groups that remain unreached and establish how these groups could be reached through secondary distribution as part of a UTT strategy.
